# The Cost-Effectiveness of Wound-Edge Protection Devices Compared to Standard Care in Reducing Surgical Site Infection after Laparotomy: An Economic Evaluation alongside the ROSSINI Trial

**DOI:** 10.1371/journal.pone.0095595

**Published:** 2014-04-18

**Authors:** Adrian Gheorghe, Tracy E. Roberts, Thomas D. Pinkney, David C. Bartlett, Dion Morton, Melanie Calvert, on behalf of the West Midlands Research Collaborative and the ROSSINI Trial Investigators

**Affiliations:** 1 Primary Care Clinical Sciences, University of Birmingham, Birmingham, United Kingdom; 2 Health Economics Unit, University of Birmingham, Birmingham, United Kingdom; 3 Academic Department of Surgery, University of Birmingham, Birmingham, United Kingdom; 4 West Midlands Research Collaborative, Birmingham, United Kingdom; National Institute for Public Health and the Environment, Netherlands

## Abstract

**Background:**

Wound-edge protection devices (WEPDs) have been used in surgery for more than 40 years to reduce surgical site infection (SSI). No economic evaluation of WEPDs against any comparator has ever been conducted. The aim of the paper was to assess whether WEPDs are cost-effective in reducing SSI compared to standard care alone in the United Kingdom.

**Methods and Findings:**

An economic evaluation was conducted alongside the ROSSINI trial. The study perspective was that of the UK National Health Service and the time horizon was 30 days post-operatively. The study was conducted in 21 UK hospitals. 760 patients undergoing laparotomy were randomised to either WEPD or standard care and 735 were included in the primary analysis. The main economic outcome was cost-effectiveness based on incremental cost (£) per quality adjusted life year (QALY) gained. Patients in the WEPD arm accessed health care worth £5,420 on average and gained 0.02131 QALYs, compared to £5,130 and 0.02133 QALYs gained in the standard care arm. The WEPD strategy was more costly and equally effective compared to standard care, but there was significant uncertainty around incremental costs and QALYs. The findings were robust to a range of sensitivity analyses.

**Conclusions:**

There is no evidence to suggest that WEPDs can be considered a cost effective device to reduce SSI. Their continued use is a waste of limited health care resources.

## Introduction

Surgical site infection (SSI) is a common postoperative complication, occurring in up to five percent (5%) of all patients undergoing surgery and 30–40% of patients undergoing abdominal surgery depending on the level of contamination [1], [2]. Development of an SSI significantly impacts upon patient mortality and morbidity as well as healthcare costs [3]. In the United Kingdom (UK), hospital length of stay is typically doubled and additional per-patient costs of up to £10,000 have been estimated, the variability depending upon the type and site of surgery and the severity of the infection [4], [5].

Wound-edge protection devices (WEPDs) have been used for more than forty years to reduce SSI by creating a physical barrier between the abdominal wound edges and viscera, visceral contents, contaminated instruments and gloves, thereby reducing accumulation of endogenous and exogenous bacteria on the wound edges. Evidence on the clinical effectiveness of WEPDs has been unclear: two systematic reviews of randomised controlled trials (RCTs) suggested that WEPD may be effective in the reduction of SSI [6], [7], although the quality of the including RCTs was low. To date there is no published evidence on the cost effectiveness of WEPDs.

The objective of the ROSSINI (Reduction of Surgical Site Infection using a Novel Intervention) trial was to explore the effectiveness and cost-effectiveness of WEPD in reducing SSI after laparotomy. In this paper we report the results of the economic evaluation conducted alongside ROSSINI which compared the relative cost-effectiveness of WEPD compared to standard care from the perspective of the UK National Health Service (NHS). The economic evaluation is reported in accordance with the CHEERS Statement (Appendix S1).

## Methods

The trial protocol (Protocol S1) and recruitment flow chart are presented as supplementary information.

### Ethics Statement

The trial protocol [8] was approved before the study began by the National Research Ethics Service (09/H1204/91; North Staffordshire Committee) and the research and development team at each hospital. Written informed consent was obtained from all patients before enrolment on paper forms approved by the aforementioned ethics body. ROSSINI was registered with controlled-trials.com (ISRCTN 40402832).

### Study Design

The full report on the trial has been reported elsewhere [9]. Briefly, the trial was a prospective, multicentre, observer blinded, randomised controlled trial with stratification according to baseline infection risk. Randomisation was performed when the patient was in the anaesthetic room immediately before surgery using a centralised secure web system provided by the University of Birmingham. Randomisation was stratified according to the urgency of surgery, likelihood of opening a viscus, and likelihood of creating a stoma, with the use of a minimisation procedure. The trial was conducted between February 2010 and January 2012 at 21 NHS hospitals across the UK. The cost-effectiveness analysis was pre-specified in the trial protocol [8]. The objective of the economic evaluation was to explore the relative cost-effectiveness of WEPD compared to standard care. Cost, resource use and outcome data in terms of QALYs were collected prospectively for both arms of the trial. Costs and QALYs for the WEPD intervention are compared to results of the standard care arm and incremental costs and incremental QALYs are calculated as the difference in costs and QALYs, respectively, between the WEPD arm and the standard care arm. When appropriate, the incremental cost-effectiveness ratio (ICER) was calculated as the ratio of incremental costs (£) to incremental effects (QALY). In this case, the ICER (£/QALY) represents the cost of obtaining an additional QALY when switching from standard care to WEPDs.

### Setting and Perspective

The trial-based evaluation took a health care provider perspective and thus considered only cost centres relevant for the NHS and Personal Social Services. The intervention under scrutiny was the use of a WEPD during surgery in addition to standard care. The comparator was no WEPD use, i.e. standard care alone. In order to enhance the generalisability of the trial, the surgical teams were given the liberty to use retraction and SSI prophylactic procedures of their choice. The time horizon was 30 days post-operatively, in accordance with SSI monitoring in the English NHS [10]. Given the short time horizon, no discounting was applied to either costs or outcomes.

### Data Collection

Health outcomes, preference-based outcomes and resource use data were collected from the participating sites using custom designed paper-based case report forms (CRFs), which were completed by patients or trial staff, as appropriate, at each site then managed centrally at the Centre for Clinical Trials at the University of Birmingham.

### Health Utility

We recorded health-related quality of life using the EuroQol EQ-5D questionnaire (the English three-level response version and validated for use in the UK, Appendix S2), a standardised generic preference based instrument that describes a patient’s health status using a single index value [11]. We used EQ-5D in this study because of its relevance for the UK policy makers, particularly the National Institute for Health and Clinical Excellence (NICE) [12]. We administered the EQ-5D instrument to patients in ROSSINI at baseline (prior to surgery) and on two occasions post-operatively. We conducted the first assessment in clinic, after the patient provided informed consent and before randomisation. We performed the second assessment (5 to 7 days) on the hospital ward if still inpatient or at discharge, as applicable. We performed the third assessment (30 to 33 days) on the hospital ward if still inpatient or, more often, in the outpatient clinic on the occasion of the scheduled follow-up visit. Only the baseline and third assessment (30 to 33 days) were used to calculate QALYs; the 5–7 days assessment was employed in the multiple imputation algorithms for missing EQ-5D scores at the final assessment.

### Resource Utilisation

We collected data on resource utilisation of health care resources prospectively in both secondary and primary care settings using the custom designed CRFs (Appendix S2). Hospital utilisation items were completed by health care staff and primary care utilisation items were completed by patients in clinic at the 30-day visit.

### Unit Costs

Unit costs were valued in £ (2011 value). The cost of the intervention (WEPD) was obtained from the manufacturer [13]. Inpatient care resource items were sourced from the NHS Reference Costs data [14], [15]. Primary care resource items were sourced from the Personal Social Services Resource Unit (PSSRU) Unit Costs and Social Care 2010–2011 [16]. Medication unit costs were taken from the British National Formulary 2011 [17]. All unit costs were average national costs. Consistent with the NHS perspective, only resource use affecting the NHS budget were considered. Total resource costs were obtained by summation of the individual resource costs for each category of resource item accessed by trial patients. Individual resource costs were obtained by multiplying the resource use by the corresponding unit costs.

### Data Analysis

In the base-case analysis we included all the patients with complete primary outcome data. Any missing cost and health utility data, as well as patient-level characteristics, were imputed using the multiple imputations using chained equations method (MICE) (Appendix S3). The analysis included descriptive statistics for the resource use items, resource costs (both at aggregate and individual level) and EQ-5D scores. QALYs were calculated by multiplying the utility weight associated with each individual health state and the time spent in that health state. QALYs were calculated based on the baseline and 30-day EQ-5D assessments and were adjusted for baseline utility [18].

The average differences in costs and outcomes, as well as the 95% confidence intervals around the point estimates, were calculated using bias-corrected and accelerated (BCa) non-parametric bootstrap with 1,000 replications [19]. The resulting incremental costs and effects were plotted on the cost-effectiveness plane, a visual decision-aiding tool representing the incremental costs and effects of the intervention under evaluation relative to the next best option [20]. Cost-effectiveness acceptability curves (CEACs) were plotted, indicating the probability of each of the two alternatives to be cost-effective at varying thresholds of the decision makers’ willingness to pay for an additional unit of outcome [21].

Sensitivity analyses were performed to check the robustness of cost-effectiveness findings, as follows: a complete case analysis based on trial subjects with complete primary outcome, cost and EQ-5D data (n = 532); and adjusted analyses for both base-case and complete case scenarios, where differences between the trial’s arms were investigated using generalized linear models. Total costs and EQ-5D scores were regressed against the following pre-specified covariates: intervention group, baseline utility (only for adjusting incremental QALYs), plan to create a stoma, plan to create a viscus (defined as any internal organ), elective/emergency surgery, age, body mass index, diabetes, current smoking status and SSI. The total cost and QALY values were regressed against the variables above using generalised linear models with an identity link [22]. A gamma distribution was assumed for costs and a normal distribution was assumed for QALYs. The analyses were performed using SAS 9.2 [23] and R 2.15.3 software [24].

## Results

ROSSINI randomised 760 patients undergoing open abdominal surgery between the use of WEPD during and standard care. 735 patients were included in the primary analysis, of which 369 patients received the intervention and 366 patients received standard care. There was no evidence of benefit for WEPDs in terms of SSI reduction (OR 0.97; 95% CI 0.69 to 1.36) or hospital length of stay [25]. In total 20 patients died within 30 days of surgery: 12 in the control group and eight in the intervention group. For those patients who died within 30 days of surgery we carried forward the last available wound assessment, unless no information was available in which case they were treated as lost to follow-up.

Table 1 presents the unit costs which informed the cost calculations. Health care utilisation data by ROSSINI patients are presented in Table 2. There is no apparent difference between the two treatment groups for neither secondary care nor primary care services. The only exception is the number of practice nurse visits: patients in the standard care arm reported twice as many practice nurse contacts than WEPD patients. When resource utilisation was aggregated as nurse visits, there were no significant differences between the two arms (Table S1).

**Table 1:** ROSSINI trial: unit costs at 2011 value.

Resource	Unit cost (£)	Source
WEPD (intervention)	15	Manufacturer [13]
HOSPITAL CARE		
Day on general ward	311	NHS Reference Costs 2010/2011 [14]
Day in ITU	1515	NHS Reference Costs 2007/2008* [15]
Day in HDU	856	NHS Reference Costs 2007/2008* [15]
PRIMARY CARE		
GP visit	36	Curtis 2011 [16]
Practice nurse visit	13	Curtis 2011 [16]
District nurse visit	73	Curtis 2011 [16]
Outpatient clinic visit	101	NHS Reference Costs 2010/2011 [14]
Medication	as appropriate	British National Formulary 2011 [17]

*The costs for a day in Intensive Therapy Unit (ITU) and a day in High Dependency Unit (HDU) were not available in NHS Reference Costs 2010/2011. The last available document where they were given explicitly was the 2007/2008 edition. For the purpose of this analysis, the 2007/2008 unit costs were updated to their 2011 value using the appropriate Hospital and community health services (HCHS) pay and price inflation (Curtis 2011).

**Table 2:** ROSSINI trial: summary of resource use by treatment group, detailed.

Resource use item	WEPD (n = 369)	Standard care (n = 366)	p-value
HOSPITAL CARE			
Inpatient days			
N	359	358	
Mean (SD)	12.55 (15.46)	11.56 (11.68)	0.3350
SE	0.82	0.62	
Median	9	9	
Days in ITU	369	366	
N	0.93 (3.12)	1.06 (5.46)	0.6913
Mean (SD)	0.16	0.28	
SE	0	0	
Median			
Days in HDU			
N	369	366	
Mean (SD)	0.60 (1.67)	0.51 (1.03)	0.6396
SE	0.09	0.08	
Median	0	0	
PRIMARY CARE			
GP visits			
N	364	358	
Mean (SD)	0.43 (0.81)	0.51 (1.03)	0.2474
SE	0.04	0.05	
Median	0	0	
District nurse visits			
N	360	355	
Mean (SD)	3.43 (7.24)	3.52 (6.94)	0.8644
SE	0.38	0.37	
Median	0	0	
Practice nurse visits			
N	366	361	
Mean (SD)	0.16 (0.70)	0.32 (1.21)	0.0355
SE	0.04	0.06	
Median	0	0	
Outpatient clinic visits			
N	364	363	
Mean (SD)	0.42 (1.09)	0.31 (0.71)	0.1205
SE	0.06	0.04	
Median	0	0	

Despite very low levels of missing data for the primary outcome, the amount of missing data for resource utilisation and health utility was somewhat higher (Table 3). EQ-5D scores at 30 days post-operatively were not available for 14% of patients, while hospital and primary care data were unavailable cumulatively for less than 10% of patients (6.66%). Overall, 20.4% of patients had incomplete observations in terms of resource use or EQ-5D data. However, there was no imbalance between the two arms with respect to the levels of missing data.

**Table 3:** ROSSINI trial: summary of missing data, by treatment group.

Missing data item	Missing observations (% of trial arm)		
	WEPD (n = 369)	Standard care (n = 366)	Trial arm differences(p-value)
HOSPITAL CARE			
Inpatient days	10 (2.7%)	8 (2.2%)	0.64
PRIMARY CARE			
GP visits	5 (1.4%)	8 (2.2%)	0.39
Practice nurse visits	3 (0.8%)	5 (1.4%)	0.47
District nurse visits	9 (2.4%)	11 (3%)	0.63
Outpatient clinic visits	5 (1.4%)	3 (0.8%)	0.48
HEALTH UTILITY			
EQ-5D data, any time point	51 (13.8%)	53 (14.5%)	0.79

### Cost-effectiveness

The base-case analysis used information from all patients with complete primary outcome data (n = 735). The results of the multiple imputation process are presented in Appendix S3. Patients in the WEPD arm accessed health care worth £5,420 on average, compared to £5,130 for patients in the standard care arm (Table 4). The use of the WEPD was associated with 0.02131 QALYs, compared to 0.02133 QALYs in the control group. Overall, the WEPD strategy was on average £290 more costly (95%CI -£372 to £948) and 0.00002 QALYs (95%CI-0.0018 to 0.0017) less beneficial than standard care, thus suggesting that WEPD was technically dominated by standard care.

**Table 4:** Costs and health utilities in the ROSSINI trial, base-case analysis.

Variable	WEPD (n = 369)	Standard care (n = 366)
**Costs (£), mean(SE)**		
Total cost	5420 (246)	5130 (234)
Intervention cost (WEPD)	15	n/a
Cost of inpatient care	5089 (247)	4812 (234)
General surgical ward	3638 (128)	3460 (122)
Intensive therapy unit	1123 (197)	1053 (186)
High dependency unit	329 (47)	299 (43)
Cost of primary care	316 (29)	318 (29)
GP visits	16 (2)	19 (2)
Practice nurse visits	2 (0.5)	4 (1)
District nurse visits	252 (27)	261 (28)
Outpatient clinic visits	44 (6)	33 (4)
Medication	1 (0.2)	1 (0.3)
**Health-related quality of life, mean (SE)**		
QALY	0.0213 (0.0014)	0.0213 (0.0014)
EQ-5D score at baseline	0.751 (0.016)	0.752 (0.016)
EQ-5D score at 30 days	0.683 (0.016)	0.684 (0.016)

Note: Standard errors (SE) were calculated assuming a Gamma distribution for costs and a normal distribution for EQ-5D scores and QALYs.

**Figure 1:**
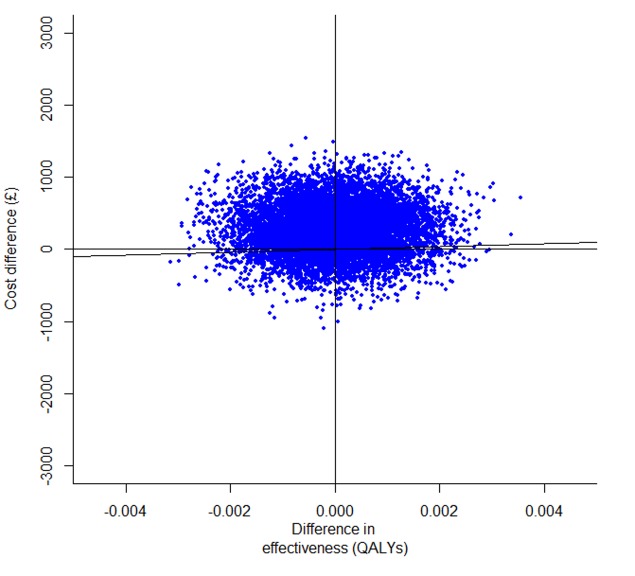
ROSSINI trial economic evaluation: cost-effectiveness plane for the base-case analysis.

**Figure 2:**
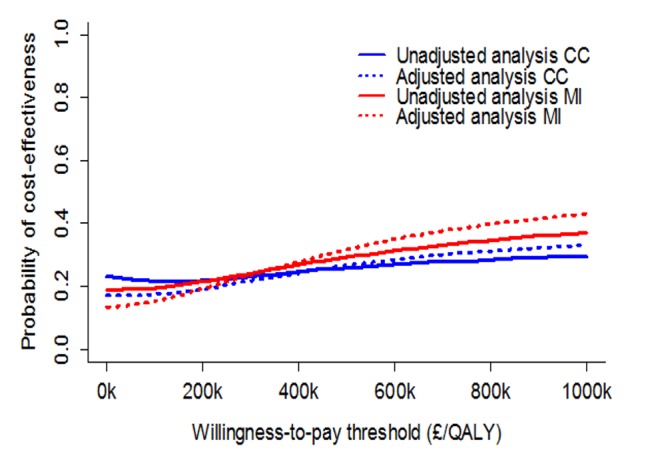
ROSSINI trial economic evaluation: comparison of cost-effectiveness acceptability curves across the analysed scenarios. Legend: MI - Base-case analysis; CC - complete case analysis.

The cost-effectiveness plane (Figure 1) shows that both incremental cost and incremental QALY estimates are associated with considerable uncertainty. The cost-effectiveness acceptability curves (CEACs) communicate the probability for an intervention to be cost-effective for a range of willingness-to-pay thresholds (£/QALY). CEACs suggest that the WEPD is less than 30% likely to be cost-effective in all analyses for the willingness-to-pay threshold range of £20,000 to £30,000 recommended by NICE (Figure 2).

### Sensitivity Analysis

The results of the regression adjusted analyses were similar to the unadjusted results: in the base-case analysis, the incremental cost increased from £290 to £311, while the QALY gain increased from −0.00002 to 0.00018 (Table 5). The resulting ICER is in excess of £1.7 million/QALY and, thus, much higher than the threshold recommended by NICE. In the unadjusted complete case scenario the ICER was £740,000/QALY; after regression adjustment the WEPD became more costly and less effective than standard care (Table 5).

**Table 5:** ROSSINI trial economic evaluation: summary of incremental costs and incremental QALYs across the analysed scenarios.

Scenario	Variable	Mean difference(WEPD – standard care)	95% BCa CI	ICER
Base-case unadjusted	Total cost (£)	290	−372 to 949	WEPD is dominated
	QALY	−0.00002	−0.0018 to 0.0017	
Base-case adjusted	Total cost (£)	311	−273 to 1012	1,712 k/QALY
	QALY	0.0002	−0.0015 to 0.0019	
Complete case unadjusted	Total cost (£)	237	−407 to 892	740 k/QALY
	QALY	0.0003	−0.0023 to 0.0016	
Complete case adjusted	Total cost (£)	369	−214 to 976	WEPD is dominated
	QALY	−0.0001	−0.0022 to 0.0019	

## Discussion

### Summary of Findings

The WEPD intervention was associated with higher costs and practically no QALY gains compared to standard care in the base-case analysis; the confidence intervals for both incremental costs and incremental QALYs show the extent of the uncertainty in the findings (Figure 1). Within the willingness-to-pay interval recommended by NICE [12], WEPDs were less than 30% likely to be cost-effective compared to standard care in all analyses (Figure 2). The result was robust to a range of sensitivity analyses. As such, WEPDs are unlikely to be cost-effective compared to standard care and their use cannot be recommended.

Although resource utilisation was comparable across the two trial arms, patients in the standard care arm appear to have received more practice nurse visits than intervention arm patients. We believe this difference may be due to mis-classification of patient reported visits on the trial case report forms (Appendix S2). A sensitivity analysis is presented in Table S1: when nurse and primary care contacts are aggregated, demonstrated that there is no significant difference between the two arms. This does not affect the cost-effectiveness findings.

### Strengths and Limitations

To our knowledge, this study is the first economic evaluation of WEPDs against any comparator and in any setting. It was an integral part of the largest multi-centre RCT to date that investigated the benefits of WEPDs. A wide range of sensitivity analyses confirmed the robustness of base-case findings.

There are also several limitations. First, the choice of time horizon may be subject to debate. The 30-day time horizon was determined by the ROSSINI primary outcome i.e. the occurrence of SSI within 30 days post-operatively, in line with the international guidelines on SSI diagnosis [10], [26]. A 30-day time horizon was also adopted in other decision models which evaluated interventions reducing SSI [27].

A further limitation refers to the complexity of SSI management, especially in primary care. NICE clinical guidelines on SSI care provide evidence that the weekly cost of wound dressings can be up to £100, depending on the type of wound and the type of dressing [28]. However, district nurses are the health care professional most likely to apply the wound dressings in a primary care setting and ROSSINI arms were more than comparable regarding the number of district nurse visits, which reduces the potential effect of not costing wound dressings (Table 2).

The uncertainty around the point estimates of incremental costs and QALYs, reflected in the width of the confidence intervals, is considerable. However, it is very unlikely that the ROSSINI trial was underpowered: the pre-specified sample size in the statistical analysis plan (n = 750), based on the best available evidence to date, assumed a 50% reduction in SSI and a 12% SSI rate in the study population. This suggests there may be a large amount of variability in the cost and QALY gains associated with the use of the WEPD. In support of this hypothesis, the primary outcome also exhibited considerable uncertainty (OR 0.97, 95% CI 0.69 to 1.36).

### Relation to other Studies

We have no knowledge of economic evaluations of WEPDs, but there are published data on SSI costs. The most recent study on the costs SSI care in a primary setting in the UK collected data on 29 SSI patients following colorectal surgery and found that primary care costs amount to about 15% of total SSI costs (on average £1,563 out of £10,523 per SSI patient), thus suggesting that the largest part of the SSI cost burden comes from inpatient care [5]. This is compatible with ROSSINI findings: primary care cost accounted for less than 10% of total costs (Table 4). However, Tanner *et al.* reported total and average resource use and costs, respectively, without any mention of the variability around these quantities. Variability is an important aspect, as illustrated in the older study of Davey *et al.* [29]: out of seven patients with a SSI in primary care, one patient alone received 57 district nurse visits, another patient received two visits and the rest no visit at all.

## Conclusion

The ROSSINI trial has shown WEPDs to be neither effective nor cost-effective in reducing SSI compared to standard care. This contradicts previous evidence, which suggested that WEPDs may be effective. ROSSINI is the largest and most robust trial investigating WEPDs to date and the first to have a pre-specified integral economic evaluation. WEPDs have been used to date at the surgeons’ discretion in the NHS to reduce SSI but there are no official data on the WEPD utilisation and, as such, the current NHS spending on WEPDs cannot be estimated. Our analyses suggest that the use of WEPDs for SSI reduction cannot be justified and should be discontinued.

## Supporting Information

Protocol S1 Trial Protocol.
(PDF)

Checklist S1 CONSORT Checklist.
(DOC)

Appendix S1 CHEERS Statement checklist for the ROSSINI within-trial economic evaluation.
(DOCX)

Appendix S2 Case report forms used to capture resource use in the ROSSINI trial.
(DOCX)

Appendix S3 Details of the multiple imputation exercise.
(DOCX)

Appendix S4 ROSSINI trial: summary of resource use by treatment group, overview.
(DOCX)
