# Supplementary material for: The Cost-Effectiveness of Wound-Edge Protection Devices Compared to Standard Care in Reducing Surgical Site Infection after Laparotomy: An Economic Evaluation alongside the ROSSINI Trial
Source: PLoS One. 2014 Apr 18;9(4):e95595. doi: 10.1371/journal.pone.0095595 (PMC3991705; doi:10.1371/journal.pone.0095595)
Supplement: Appendix S2 — Case report forms used to capture resource use in the ROSSINI trial. (DOCX) [file pone.0095595.s004.docx]

**Appendix 2. Case report forms used to capture resource utilisation in the ROSSINI trial**

**ROSSINI – Reduction of Surgical Site Infection using a Novel Intervention**

**CRF 4 – Wound healing post-discharge Questionnaire - 30-33 days**

**(Version 4.0 dated 14th March 2011)**

**To be completed by Patient**

| **ROSSINI trial number** |  | | |  | | | | |
| --- | --- | --- | --- | --- | --- | --- | --- | --- |
|  |  | | | | | | | |
| **Centre name** |  | | | | | | | |
|  |  | | | | | | | |
| **Date of birth** | **d** | **d** | **m** | **m** | **y** | **y** | **y** | **y** |
|  |  | | | | | | | |
| **Date of operation** | **d** | **d** | **m** | **m** | **y** | **y** | **y** | **y** |
|  |  |  |  |  |  |  |  |  |
| **Date form completed** | d | d | m | m | y | y | y | y |
|  |  |  |  |  |  |  |  |  |

Hospital Sticker

Since you were discharged from hospital after your operation have you noticed any of the following?

| 1 | Was there any **discharge** or **leakage** from any part of the wound? | | | | |  | | | Yes | | | | |  | | | No | | | | |  | | |
| --- | --- | --- | --- | --- | --- | --- | --- | --- | --- | --- | --- | --- | --- | --- | --- | --- | --- | --- | --- | --- | --- | --- | --- | --- |
|  |  | | |  | |  | | |  | | | | |  | | |  | | | | |  | | |
|  | If YES was it | | | Clear or Blood-stained |  |  | | |  | | | | |  | | |  | | | | |  | | |
|  |  | | |  | |  | | |  | | | | |  | | |  | | | | |  | | |
|  |  | | | Yellow/Green (pus) |  |  | | |  | | | | |  | | |  | | | | |  | | |
|  |  | | |  | |  | | |  | | | | |  | | |  | | | | |  | | |
|  |  | | | Other – please specify |  |  | | |  | | | | |  | | |  | | | | |  | | |
|  |  | | | | |  | | |  | | | | |  | | |  | | | | |  | | |
| 2 | Did any health care worker take a sample or swab from your wound and send it to the laboratory? | | | | |  | | | Yes | | | | |  | | | No | | | | |  | | |
|  |  | | | | |  | | |  | | | | |  | | |  | | | | |  | | |
| 3 | Please tick any of the following that applied to your wound: | | | | |  | | |  | | | | |  | | |  | | | | |  | | |
|  |  | | | | |  | | |  | | | | |  | | |  | | | | |  | | |
|  |  | **Pain** or **soreness** in addition to the discomfort experienced following the operation | | | |  | | |  | | | | |  | | |  | | | | |  | | |
|  |  |  | | | |  | | |  | | | | |  | | |  | | | | |  | | |
|  |  | **Redness** or **inflammation** spreading from the edges of the wound | | | |  | | |  | | | | |  | | |  | | | | |  | | |
|  |  |  | | | |  | | |  | | | | |  | | |  | | | | |  | | |
|  |  | The area around the wound felt **warmer** than the surrounding skin | | | |  | | |  | | | | |  | | |  | | | | |  | | |
|  |  |  | | | |  | | |  | | | | |  | | |  | | | | |  | | |
|  |  | The area around the wound become **swollen** | | | |  | | |  | | | | |  | | |  | | | | |  | | |
|  |  |  | | | |  | | |  | | | | |  | | |  | | | | |  | | |
|  |  | The edges of any part of the wound **separated or gaped open** | | | |  | | |  | | | | |  | | |  | | | | |  | | |
|  |  | |  | | |  | | |  | | | | |  | | |  | | | | |  | | |
| 4 | Please tell us the date you noticed these symptoms  (If you cannot remember the exact date, please estimate) | | | | |  |  | | | |  | |  | | |  | | |  | |  | | |  |
|  |  |  |  |  |  | d | | d | | m | | m | | | y | | | y | | y | | | y | |

**Please continue overleaf**

| **ROSSINI trial number** |  | | |  | | | | |
| --- | --- | --- | --- | --- | --- | --- | --- | --- |
|  |  | | | | | | | |
|  |  | | | | | | | |
| **Date of birth** | **d** | **d** | **m** | **m** | **y** | **y** | **y** | **y** |
|  |  | | | | | | | |

|  |  |  | | | | | |  | |  |  | |  |  |
| --- | --- | --- | --- | --- | --- | --- | --- | --- | --- | --- | --- | --- | --- | --- |
| 5a |  | Have you been given antibiotics for an infection in the wound? | | | | | | Yes | |  | No | |  |  |
|  |  |  | | | | | | | | | | | | |
|  |  | Please specify name of antibiotic and dose (if possible) and who gave you them | | | | | | | | | | | | |
|  |  |  | | | | | |  | |  |  | |  |  |
| 5b |  | Have you received a prescription from your GP for pain relief? | | | | | | Yes | |  | No | |  |  |
|  |  |  | | | | | |  | |  |  | |  |  |
|  |  | If so, please specify name of drug and dose (if possible) | | | | | | | | | | | | |
|  |  |  | | | | | |  |  | |  |  | |  |
| 6 |  | Since your operation have you visited any of the following in relation with your wound? | | | | | |  |  | |  |  | |  |
|  | | | Your GP? | Yes |  | No |  |  | If YES, number of visits | | | | |  |
|  | | |  |  |  |  |  |  |  | | | | |  |
|  | | | A practice nurse? | Yes |  | No |  |  | If YES, number of visits | | | | |  |
|  | | |  |  |  |  |  |  |  | | | | |  |
|  | | | Outpatient clinic at the hospital? | Yes |  | No |  |  | If YES, number of visits | | | | |  |
|  | | |  |  |  |  |  |  |  | | | | |  |
| Have you been visited by a district nurse: | | | | Yes |  | No |  |  | If YES, number of visits | | | | |  |

| 7 Have you been re-admitted to hospital with an infection of the surgical wound? |  | Yes |  | No |  |
| --- | --- | --- | --- | --- | --- |

If YES, which Hospital? __________________________________

How many nights were you an inpatient?

8 Please provide any other information about the healing of your wound:

________________________________________________________________________________________________________________________________________________________________________________

Many thanks.

**ROSSINI – Reduction of Surgical Site Infection using a Novel Intervention**

**CRF 4a – Wound healing diary**

**(Version 2.0 dated 14th March 2011)**

**To be completed by Patient**

| **ROSSINI trial number** |  | | |  | | | | | |
| --- | --- | --- | --- | --- | --- | --- | --- | --- | --- |
|  |  | | | | | | | | |
| **Centre name** |  | | | | | | | | |
|  |  | | | | | | | | |
| **Date of birth** | d | d | m | | m | y | y | y | y |
|  |  | | | | | | | | |
| **Date of operation** | d | d | m | | m | y | y | y | y |
|  |  |  |  | |  |  |  |  |  |
| **Date form to be returned** | d | d | m | | m | y | y | y | y |
|  |  |  |  | |  |  |  |  |  |

Hospital Sticker

| d | d | m | m | y | y | y | y |
| --- | --- | --- | --- | --- | --- | --- | --- |

**Date today**

|  | |  | | | | | | | | | | | |  | | | |  | | |  | | | |  |
| --- | --- | --- | --- | --- | --- | --- | --- | --- | --- | --- | --- | --- | --- | --- | --- | --- | --- | --- | --- | --- | --- | --- | --- | --- | --- |
| 1 | | Has your wound infection got better? | | | | | | | | | | | | Yes | |  | | | No | | | |  | | |
|  | | If Yes, approximate date | d | d | m | m | | y | | y | | y | y |  | |  | | |  | | | |  | | |
|  | |  | | | | | | | | | | | |  | |  | | |  | | | |  | | |
| 2 | | Have you been given **antibiotics** for an infection in the wound within the last month? | | | | | | | | | | | | Yes | |  | | | No | | | |  | | |
|  | |  | | | | | | | | | | | |  | |  | | |  | | | |  | | |
|  | | Please specify name of antibiotics and dose (if possible) and who gave you them | | | | | | | | | | | | | | | | | | | | | | | |
|  | |  | | | | | | | | | | | | | | | | | | | | | | | |
| 3 | | Since your operation have you visited any of the following in relation with your wound?: | | | | | | | | | | | | | | | | | | | | | | | |
|  | |  | | | | | | | | | | | | | | | | | | | | | | | |
| Your GP? | | | | | No | |  | | Yes | |  | | If YES, number of visits | | | | | | | | |  | | | |
|  | | | | |  | |  | |  | |  | |  | | | | | | | | |  | | | |
| Practice nurse? | | | | | No | |  | | Yes | |  | | If YES, number of visits | | | | | | | | |  | | | |
|  | | | | |  | |  | |  | |  | |  | | | | | | | | |  | | | |
| Outpatient clinic at the hospital? | | | | | No | |  | | Yes | |  | | If YES, number of visits | | | | | | | | |  | | | |
|  | | | | |  | |  | |  | |  | |  | | | | | | | | |  | | | |
| Have you been visited by a district nurse? | | | | | No | |  | | Yes | |  | | If YES, number of visits | | | | | | | | |  | | | |
|  |  | | | | | | | | | | | | | | | | | | | | | | | | |
|  |  | | | | | | | | | | | | | |  | |  | | |  | | | |  | |
| 4 | Have you been re-admitted to hospital with an infection of the surgical wound? | | | | | | | | | | | | | | Yes | |  | | | No | | | |  | |

If yes, which Hospital? ___________________________________________________

How many nights were you an inpatient?

| 5 | Please provide any other information about the healing of your wound: |
| --- | --- |

Many thanks.

**ROSSINI – Reduction of Surgical Site Infection using a Novel Intervention**

**CRF 6 – Resource Utilisation (up to 30 days) Version 3.0 dated 14th March 2011**

**Patient Information**

Hospital Sticker

| **ROSSINI trial number** |  | | |  | | | | |
| --- | --- | --- | --- | --- | --- | --- | --- | --- |
|  |  | | | | | | | |
| **Centre name** |  | | | | | | | |
|  |  |  |  |  |  |  |  |  |
| **Date of birth** | **d** | **d** | **m** | **m** | **y** | **y** | **y** | **y** |
|  |  | | | | | | | |
| **Date form completed** | **d** | **d** | **m** | **m** | **y** | **y** | **y** | **y** |
|  |  |  |  |  |  |  |  |  |

**Person completing form**

**HOSPITAL CARE: INITIAL OPERATION**

| Date of Admission |  | | | | | | | | Date of Surgery |  | | | | | | | |  |
| --- | --- | --- | --- | --- | --- | --- | --- | --- | --- | --- | --- | --- | --- | --- | --- | --- | --- | --- |
|  | d | d | m | m | y | y | y | y |  | d | d | m | m | y | y | y | y | |

***Please tick one box only***

|  |  | Discharged |  | Died in hospital | | | | |  | | Still inpatient at time of this review | | |  |  |
| --- | --- | --- | --- | --- | --- | --- | --- | --- | --- | --- | --- | --- | --- | --- | --- |
|  |  |  |  |  | | | | |  | |  | | | | |
| Date of death or discharge | | | | |  |  |  |  |  |  |  |  |  |  |  |
|  |  |  |  |  | d | d | m | m | y | y | y | Y |  |  |  |

|  |  |  |
| --- | --- | --- |

| Date of Re-Admission |  | | | | | | | |
| --- | --- | --- | --- | --- | --- | --- | --- | --- |
|  | d | d | m | m | y | y | y | y |

**READMITTED TO HOSPITAL?**

***Please tick one box only***

|  |  | Discharged | |  | | Died in hospital | | | | | | |  | Still inpatient at time of this review |  |
| --- | --- | --- | --- | --- | --- | --- | --- | --- | --- | --- | --- | --- | --- | --- | --- |
|  |  |  | |  | |  | | |  | | | | |  |  |
| Date of death or discharge if readmitted | | |  | |  | |  |  |  |  |  |  |  |  |  |
|  |  |  | d | | d | | m | m | y | y | y | Y |  |  |  |

**Within 30 day period post operation, please specify:**

| Number of nights in critical care (ICU or HDU) |  |  |
| --- | --- | --- |
|  |  |  |
|  |  |  |
| Number of nights on ventilator |  |  |
|  |  |  |

**PLEASE CONTINUE OVERLEAF**

| **ROSSINI trial number** |  | | |  | | | | |
| --- | --- | --- | --- | --- | --- | --- | --- | --- |
|  |  | | | | | | | |
|  |  | | | | | | | |
| **Date of birth** | **d** | **d** | **m** | **m** | **y** | **y** | **y** | **y** |
|  |  | | | | | | | |

**ANTIBIOTIC USEAGE**

| **Antibiotic name** | **Daily Dose** | | **Date Started** | | | | | | | | **Date Stopped** | | | | | | | | **Delivered** |
| --- | --- | --- | --- | --- | --- | --- | --- | --- | --- | --- | --- | --- | --- | --- | --- | --- | --- | --- | --- |
|  | Total daily dose | Route |  |  |  |  |  |  |  |  |  |  |  |  |  |  |  |  | Induction  On Ward  Post discharge? |
|  |  |  |  |  |  |  |  |  |  |  |  |  |  |  |  |  |  |  |  |
|  |  |  | d | d | m | m | y | y | y | y | d | d | m | m | y | y | y | y |  |
|  |  |  |  |  |  |  |  |  |  |  |  |  |  |  |  |  |  |  |  |
|  |  |  | d | d | m | m | y | y | y | y | d | d | m | m | y | y | y | y |  |
|  |  |  |  |  |  |  |  |  |  |  |  |  |  |  |  |  |  |  |  |
|  |  |  | d | d | m | m | y | y | y | y | d | d | m | m | y | y | y | y |  |
|  |  |  |  |  |  |  |  |  |  |  |  |  |  |  |  |  |  |  |  |
|  |  |  | d | d | m | m | y | y | y | y | d | d | m | m | y | y | y | y |  |
|  |  |  |  |  |  |  |  |  |  |  |  |  |  |  |  |  |  |  |  |
|  |  |  | d | d | m | m | y | y | y | y | d | d | m | m | y | y | y | y |  |
|  |  |  |  |  |  |  |  |  |  |  |  |  |  |  |  |  |  |  |  |
|  |  |  | d | d | m | m | y | y | y | y | d | d | m | m | y | y | y | y |  |

**OTHER TREATMENT:**

**Pain relief whilst inpatient**

| Number of days epidural |  |
| --- | --- |
|  |  |
| Number of days PCA |  |

**Other analgesia given (regular or prn)**

| **Name of drug** | **Dose** | **Number of administrations** |
| --- | --- | --- |
|  |  |  |
|  |  |  |
|  |  |  |
|  |  |  |
|  |  |  |
|  |  |  |
|  |  |  |
|  |  |  |
|  |  |  |
|  |  |  |

**Pain relief prescribed at either discharge (after initial operation AND readmission, if applicable)**

| **Name of drug** | **Dose** | **Quantity dispensed** |
| --- | --- | --- |
|  |  |  |
|  |  |  |
|  |  |  |
