# Supplementary material for: The Cost-Effectiveness of Wound-Edge Protection Devices Compared to Standard Care in Reducing Surgical Site Infection after Laparotomy: An Economic Evaluation alongside the ROSSINI Trial
Source: PLoS One. 2014 Apr 18;9(4):e95595. doi: 10.1371/journal.pone.0095595 (PMC3991705; doi:10.1371/journal.pone.0095595)
Supplement: Appendix S3 — Details of the multiple imputation exercise. (DOCX) [file pone.0095595.s005.docx]

**Appendix 3. Details of the multiple imputation exercise**

Missing resource costs and health utilities were imputed using MICE methods [[1](#_ENREF_1)], where missing data are imputed based on the conditional density of a variable in relation to the other variables and it is assumed that the data are missing at random. The imputations were performed using the *mice* package available in R 2.15.3 software [[2](#_ENREF_2)]. Resource costs and EQ-5D scores were imputed using an algorithm which predicted the missing values based on a wide range of variables: patient and operative characteristics (age, BMI, smoking status, diabetes, plan to open viscus, plan to create a stoma, elective/emergency surgery, ASA grade, duration of surgery, SSI status); hospital care cost items; primary care cost items; and EQ-5D scores at baseline, at 5-7 days post-operatively and at 30-33 days post-operatively. The predictive mean matching method was used to impute patient-level characteristics. Costs were bounded to be positive and EQ-5D scores were bounded between -0.594 and 1, in accordance with the UK scoring algorithm [[3](#_ENREF_3)]. Twenty datasets (each obtained after 20 iterations) were generated from the imputation process. For the adjusted analysis, regression coefficients and their variances were combined using Rubin's rules [[4](#_ENREF_4)].

The diagnosis of the multiple imputation process is presented below. The imputation sets appear to converge for all the variables and no trend is apparent, which suggests that the results of the imputation can be used in confidence [[5](#_ENREF_5)].


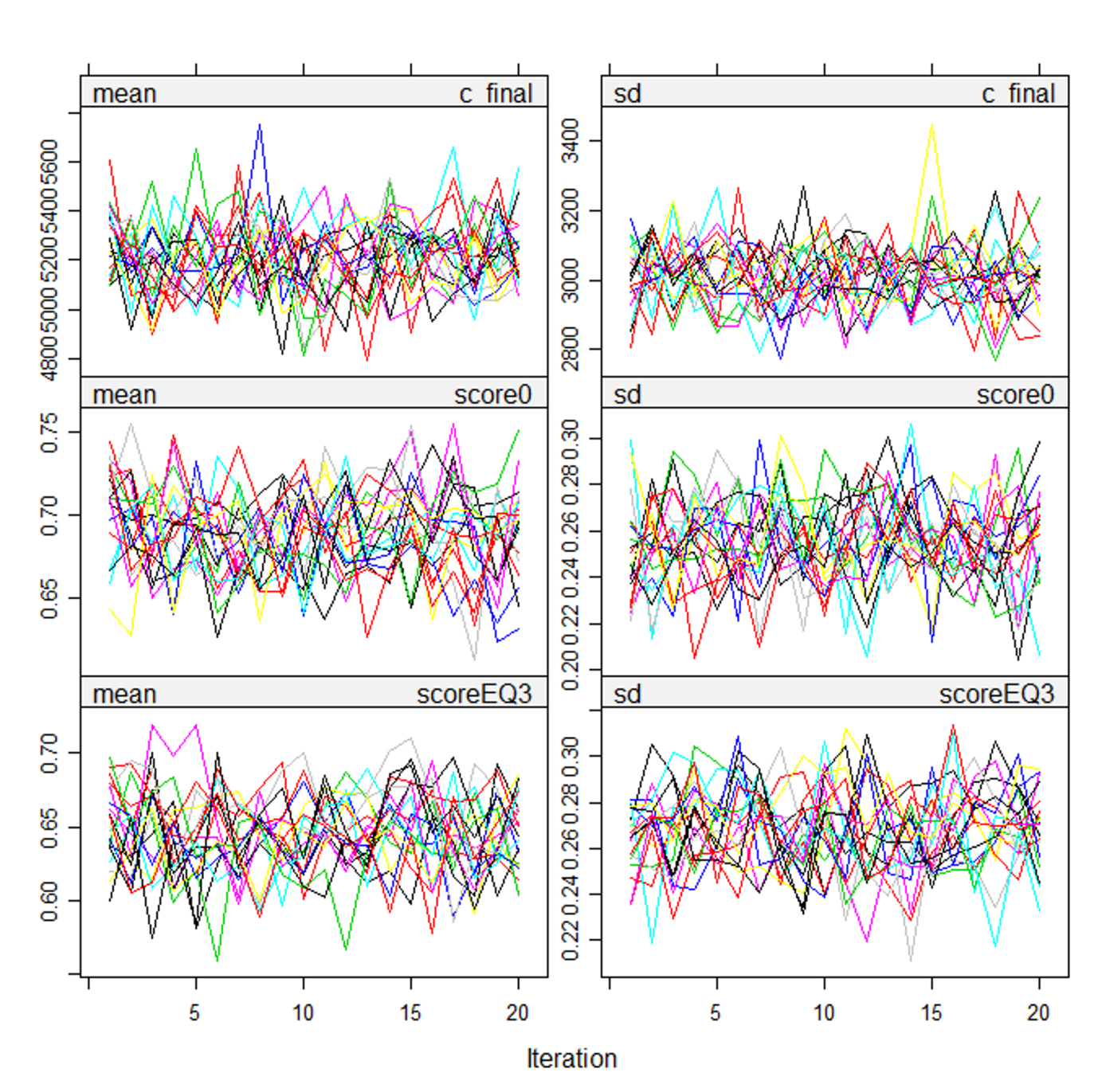


Note: Depicted variables are: total cost (c_final), baseline EQ-5D (score0) and final EQ-5D (scoreEQ3). For each variable the results of the multiple imputation exercise (20 sets) across 20 iterations (x axis) are depicted for the mean and standard deviation.

References

White IR, Royston P and Wood AM (2011) Multiple imputation using chained equations: Issues and guidance for practice. Statistics in Medicine 30: 377-399.

van Buuren S and Groothuis-Oudshoorn K (2013) Package 'mice' Multivariate Imputation by Chained Equations.

Dolan P (1997) Modeling Valuations for EuroQol Health States. Medical Care 35: 1095-1108.

Rubin DB (1987) Multiple Imputation for Nonresponse in Surveys. New York: Wiley.

Van Buuren S and Groothuis-Oudshoorn K (2011) mice: Multivariate Imputation by Chained Equations in R. Journal of Statistical Software 45.
