# Supplementary material for: The Cost-Effectiveness of Wound-Edge Protection Devices Compared to Standard Care in Reducing Surgical Site Infection after Laparotomy: An Economic Evaluation alongside the ROSSINI Trial
Source: PLoS One. 2014 Apr 18;9(4):e95595. doi: 10.1371/journal.pone.0095595 (PMC3991705; doi:10.1371/journal.pone.0095595)
Supplement: Appendix S4 — ROSSINI trial: summary of resource use by treatment group, overview. (DOCX) [file pone.0095595.s006.docx]

**Appendix 4. ROSSINI trial: summary of resource use by treatment group, overview**

| Resource use item | WEPD (n=369) | Standard care (n=366) | p-value |
| --- | --- | --- | --- |
| HOSPITAL CARE |  |  |  |
| Inpatient days | | | |
| N  Mean (SD)  SE  Median | 359  12.55 (15.46)  0.82  9 | 358  11.56 (11.68)  0.62  9 | 0.3350 |
| PRIMARY CARE |  |  |  |
| Primary care points of contact (includes GP visits, all nurse visits and outpatient clinic visits) | | | |
| N  Mean (SD)  SE  Median | 350  4.38 (7.59)  0.41  1 | 347  4.47 (7.02)  0.38  2 | 0.8795 |
| Nurse visits (includes district nurse visits and practice nurse visits) | | | |
| N  Mean (SD)  SE  Median | 357  3.54 (7.16)  0.38  0 | 352  3.74 (6.81)  0.36  0 | 0.6939 |
| The above section suggests that a large number of patients did not report any primary care visits (median is 0). The section below only looks at patients who reported at least one primary care visit (GP, practice nurse, district nurse or outpatient clinic) and at least one nurse visit, respectively. | | | |
| Primary care points of contact (includes GP visits, all nurse visits and outpatient clinic visits) | | | |
| N  Mean (SD)  SE  Median | 242  6.88 (8.57)  0.57  3 | 247  6.80 (7.70)  0.51  3 | 0.9163 |
| Nurse visits (includes district nurse visits and practice nurse visits) | | | |
| N  Mean (SD)  SE  Median | 188  7.18 (8.83)  0.66  4 | 189  7.54 (8.04)  0.61  4 | 0.6937 |
